# A clinical decision support system in back pain helps to find the diagnosis: a prospective correlation study

**DOI:** 10.1007/s00402-021-04080-y

**Published:** 2021-08-04

**Authors:** Achim Benditz, Loreto C. Pulido, Joachim Grifka, Fabian Ripke, Petra Jansen

**Affiliations:** 1 Department of Orthopaedics, University Medical Centre Regensburg, Asklepios Klinikum Bad Abbach, Kaiser-Karl-V-Allee 3, 93077 Bad Abbach, Germany; 2 Allianz Digital Health GmbH, Willy-Brandt-Platz 3, 81829 München, Germany; 3 Department of Sport Science, University of Regensburg, Universitaetsstraße 31, 93053 Regensburg, Germany

**Keywords:** Decision support system, Back pain, Symptom checker, App-based decision

## Abstract

The aim of this study is to show the concordance of an app-based decision support system and the diagnosis given by spinal surgeons in cases of back pain. 86 patients took part within 2 months. They were seen by spine surgeons in the daily routine and then completed an app-based questionnaire that also led to a diagnosis independently. The results showed a Cramer’s *V* = .711 (*p* < .001), which can be taken as a strong relation between the tool and the diagnosis of the medical doctor. Besides, in 67.4% of the cases, the diagnosis was concordant. An overestimation of the severity of the diagnosis occurred more often than underestimation (15.1% vs. 7%). The app-based tool is a safe tool to support healthcare professionals in back pain diagnosis.

## Introduction

Back pain is one of the leading reasons for people seeking for health care services [1]. Thus, back pain is a serious disease of increasing socio-economic importance [2–5]. Globally, especially low back pain is the main cause of years lived with disability [3]. Every age group is affected by back pain. The incidence of low back pain is 60–90%, and low back pain is the main cause of working disability in most countries [6–10]. Also neck pain is in increasing problem with incidences of between 10.4% and 71.5%, and an annual prevalence varying between 30 and 50% [7, 11–16]. Because of these enormous costs for the health care system, more and more attempts are made to support health care providers with a computerized decision support systems (DSS). The DSS can be considered as one specific form of a symptom checker [10]. The study group of the authors has already developed and published a first use of a computerized algorithm which is based on the next best questions to find out the most possible diagnosis [10]. Also, other institutions use these systems. Already in 2015, Semigran et al. examined 23 symptom checkers for self-diagnoses and triage using 45 standardized patients. The results showed that overall the correct diagnosis was given in 34% of the patients and the correct diagnosis under the top 20 diagnoses was given in 58% [17]. A study in 2018 showed significant medium correlation between the DSS and the medical recommendation. In 49.6% the diagnoses were concordant [10].

The aim of the present study was to redesign the questions and algorithm to improve the concordance between medical diagnosis given by a spinal surgeon and the algorithm tool.

## Methods

This non-randomized unblinded correlational study included male and female patients with back pain who visited the Department of Orthopaedics of the University Medical Centre Regensburg between September and November 2020. Inclusion criteria were: consultation because of back pain, German language to understand the questions. Exclusion criteria were missing consent or patients who were not able to take part because of other medical reasons. The study was approved by the Ethics Commission of the University of Regensburg (21.08.18, 18-1007-121, DRKS DRKS00012467) and carried out in accordance with the approved guidelines of the Helsinki Declaration of 1975. A written informed consent was obtained from all study participants. Participation in this study was voluntary.

## Patients

After fulfilling the inclusion criteria, 89 patients were included, of whom 86 completed the study. Three patients had to be excluded due to technical problems, so they were not able to complete the questionnaire. From the remaining 86 patients, 40 were female and 46 males. (Table 1).

**Table 1:** Information on age, weight and height for women and men

	Women (*N* = 40)	Men (*N* = 46)
Age (mean (SD), Min–Max)	50.90 (± 16.83), 21–84	49.39 (15.89), 19–77
Weight (mean (SD), Min–Max)	75.13 (20.23), 52–160	88.96 (14.57), 54–123
Height (mean, (SD), Min–Max)	164.38 (17.71), 62–184	179.65 (8.21), 162–198

### App-based questionnaire for back pain

The algorithm of the app-based tool was developed in cooperation with members of Digital Health, medical doctors and psychologists. The idea for the tool algorithm is an algorithm that reflects the spinal surgeons´ way of thinking while he is taking the anamneses. First the algorithm was written down as a big decision tree. Then an app-based question tool was taken, and the questions were used within this frame setting to facilitate the evaluation. Thus, the algorithm and not the tool itself was subject of investigation.

For safety reasons the typical red flag questions have to be answered all with “no”, otherwise the patient is sent directly to an emergency room. Then the patient chooses the area of pain (cervical, thoracic, lumbar, S-I-Joint).

After that two to five dichotomic questions have to be answered with yes or no. These “decision” questions lead to the most likely diagnosis. This diagnosis is then reassured by a block of questions, which are sensitive for the supposed diagnosis. If more than 65% of the answers are “yes”, the diagnosis is confirmed. If not, the second most likely diagnosis is evaluated, by again a block of sensitive questions.

If these are answered with “yes” in more than 65%, this diagnosis is taken, if not the patient it advised to go to see a doctor because a diagnosis cannot be found for certain (“no diagnosis”). See in Table 2 an overview of all possible diagnoses.

**Table 2:** Overview of possible diagnoses in the algorithm

Buttock	Lumbar spine	Thoracic spine	Cervical spine	Whole spine
Iliosacral joint block Sacroilitis	Ankylosing spondylitis Facet joint arthritis Herniated disc Low back pain Spondylodiscitis Osteoporotic vertebra fracture Spinal stenosis Spondylolisthesis	Ankylosing spondylitis Osteoporotic vertebra fracture Spondylodiscitis Thoracic block	Cervicocephalic syndrome Herniated disc Myelogelosis Spinal stenosis	Fibromyalgia Psychologically induced back pain

### Procedure

The study was conducted in the Department of Orthopaedics of a University Medical Centre. All patients were seen by one of two spinal surgeons. Each examination took between 10 and 20 min. The app-based form was filled either before the appointment or directly before the consultation while waiting by the patient itself within a maximum of 5 min. The order was chosen by chance for a smooth integration in the daily clinical routine. There were two experienced consultant spine surgeons of the same experience working together for 10 years, which means that their thinking and decisions are comparable. They were blinded in that way, that they did not have any knowledge of the result of the tool. Thus, both procedures (tool and medical examination) were treated independently.

After a first pilot study of 20 patients, the technical setting was improved using a better tablet. Some mistakes in linking the questions within the algorithm were fixed and the present study started.

### Statistical analysis

To calculate the number of necessary cases, the effect size was taken from our former study [10]: With an effect size of phi = 0.717, an error probability of 5% and a power of 95%, the calculated number of cases is 86 [18].

The scored diagnoses were given by the spinal surgeons manually and by the tool as saved data. At first, the correlation between the diagnoses of the tool and the medical diagnosis were calculated. Because data were nominal scaled, and both variables have more than two characteristics, Cramers *V* was used. All diagnoses were described in the result section. Furthermore, the combination between the medical and the computerized diagnoses were presented for every single diagnosis. A possible difference in the frequency was statistically investigated with the Chi-square test.

“Overestimation” means, that the tool´s diagnosis is more severe than the spinal surgeon´s diagnosis. E.g., the tool says herniated disc and the spinal surgeon says unspecific low back pain. The diagnoses facet joint arthritis, low back pain and Iliosacral joint block were regarded as equal as there is nearly the same first line conservative treatment.

## Results

Relation between the tool and the diagnosis of the spinal surgeon:

Table 3 shows descriptively the relation of the single diagnoses given by the tool and the medical doctor.

**Table 3:** Combination of single diagnoses from the tool and the spinal surgeon; each number represents one diagnose and you can see how often tool and how often the spine surgeons diagnosed it; e.g. the diagnosis spinal stenosis (2) was set ten times by the spine surgeons, whereas the tool had this result eight times and one facet joint arthritis (1) and one result was “no diagnosis” (12)

	Diagnosis tool												
	1	2	3	4	5	6	7	8	9	11	12	13	Total
Diagnosis spine surgeon													
1	3	0	0	2	0	0	5	0	0	0	0	0	10
2	1	8	0	0	0	0	0	0	0	0	1	0	10
3	0	2	8	1	0	0	0	0	1	0	3	0	15
4	1	0	0	3	0	0	3	0	0	0	2	0	9
5	0	0	0	0	1	0	0	0	0	0	0	0	1
6	0	0	0	0	0	13	0	0	0	0	0	0	13
7	0	0	0	0	0	0	10	0	0	0	0	0	10
8	0	1	0	0	0	0	1	4	0	0	2	2	10
10	4	0	0	0	1	0	0	0	1	0	0	0	6
11	0	0	0	0	0	0	0	0	0	1	0	0	1
13	0	1	0	0	0	0	0	0	0	0	0	0	1
Total	9	12	8	6	2	13	19	4	2	1	8	2	86

1 = facet joint arthritis, 2 = spinal stenosis, 3 = herniated disc, 4 = low back pain, 5 = osteoporotic vertebra fracture, 6 = Iliosacral joint block, 7 = thoracic block, 8 = cervical myelogelosis, 9 = spondylodiscitis, 10 = spondylolisthesis, 11 = fibromyalgia, 12 = no diagnosis, 13 = cervicocephalic syndrome

Statistically, a significant relation between the tool and the diagnosis of the medical doctor could be carved out, Cramer’s *V* = 0.711, *p* < 0.001, which can be taken as a strong relationship. This relation holds true, if the data were calculated separately for women, Cramer’s *V* = 0.720, *p* < 0.001 and men, Cramer’s *V* = 0.780, *p* < 0.001.

In 67.4% of the cases the diagnosis between the medical doctors and the DSS were concordant, in 32.6% they were discordant. The difference of the frequencies was statistically significant, *χ*^2^ (1, *N* = 86) = 10.47, *p* < 0.001).

Analysing the over- and underestimation (fusing the diagnoses: facet joint arthritis, low back pain and Iliosacral joint block) the results showed that in 77.9% of the cases the diagnoses were concordant, in 15.1% they were overestimated, and in 7% they were underestimated by the DSS. The frequencies of the categories were statistically significant, *χ*^2^ (2, *N* = 86) = 77.74, *p* < 0.001); however, the difference of the frequencies between the categories “overestimation” and “underestimation” was not significant, *χ*^2^ (1, *N* = 19) = 2.58, *p* = 0.108).

## Discussion

The hypothesis that there is a correlation between the diagnoses of the spinal surgeons and the clinical decision support tool holds true. In comparison to former studies, the correlational effects are strong.

Since ages symptom checkers are part of scientific work with the aim to unify and to simplify clinical diagnosis finding or to support patients identifying their problems themselves [19, 20]. In addition, the consortium selfBACK introduced a protocol to be used by patients themselves to promote self-management of low back pain (LBP) [21, 22].

Despite all these efforts only few studies were published in the field of back pain in the last years. In the present study 67.4% of the diagnoses between the medical doctors and the DSS were concordant, analysing the over- and underestimation the results even showed that 77.9% of the diagnoses were concordant.

That means that e.g., pain because facet joint arthritis or low back pain was counted equally. Only 7% of the diagnoses were underestimated by the patients. But none of them was an urgent case. E.g., the tool recognized low back pain and the spinal surgeons diagnosed a spondylolisthesis without surgical indication.

In comparison to other studies the concordance is really strong. In 2015 Semigran et al. evaluated 23 symptom checkers for self-diagnoses and triage [17]. For this evaluation 45 standardized patient vignettes were formed. 15 were each assigned to a group. Group 1 needed immediate help, group 2 need no immediate help and group 3 only required exercises on their own. Overall in the 23 symptom checkers, only in 34% the correct diagnosis was given [17]. In another study by Bison et al., the patients filled the symptom checker and then identify the cause of their knee pain themselves in a list of 2–15 diagnoses. This only worked in 58% of the cases [23].

Further aim of this should not be to replace the medical doctor, but to help patients to have an initial assessment of their symptoms, decision making in outpatient centres or patient telephone hotline. In addition, it could help to triage in emergency rooms. The assessment only takes about 5 min. So, the tool can be used in waiting rooms as stand-alone tablet or on telephone hotlines by medical staff.

Due to the different treatment methods and individual decisions, treatment recommendations were deliberately avoided when developing this tool. However, diagnosis-specific physiotherapy exercises are recommended to patients.

In our former study, it is noticeable that there was a minor number of psychiatric diagnoses, a result which was not in line with the literature because depression and anxiety is related to back pain as well as avoidance behaviour [10, 17, 24]. In the algorithm of this study, only chronic pain syndrome and fibromyalgia are possible diagnoses.

Despite the growing digitalization in all fields, this study is the first for 2 years to evaluate a specific kind of symptom checker for back pain.

## Limitations

There are some limitations to the study. Only spinal surgeons who work at the same hospital were included for the clinical examination, which means that most patients have had some treatment before. Next, outpatient centres should be included, as there is usually the first contact to the patient and the doctors are general practitioners and not spine surgeons. Another limitation is that psychiatric diagnoses are not evaluated.

## Conclusion

This study showed strong correlation between the diagnoses by spinal surgeons and the clinical decision support system in back pain patients. In a next step it should be evaluated, if the tool can help to reduce the time that passes before a successful treatment is achieved for those patients.

## Data Availability

The data used to support the findings of this study are available from the corresponding author upon request.
